# Efficient Conversion of Phenylpyruvic Acid to Phenyllactic Acid by Using Whole Cells of *Bacillus coagulans* SDM

**DOI:** 10.1371/journal.pone.0019030

**Published:** 2011-04-20

**Authors:** Zhaojuan Zheng, Cuiqing Ma, Chao Gao, Fengsong Li, Jiayang Qin, Haiwei Zhang, Kai Wang, Ping Xu

**Affiliations:** 1 State Key Laboratory of Microbial Technology, Shandong University, Jinan, People's Republic of China; 2 MOE Key Laboratory of Microbial Metabolism and School of Life Sciences and Biotechnology, Shanghai Jiao Tong University, Shanghai, People's Republic of China; New England Biolabs, Inc., United States of America

## Abstract

**Background:**

Phenyllactic acid (PLA), a novel antimicrobial compound with broad and effective antimicrobial activity against both bacteria and fungi, can be produced by many microorganisms, especially lactic acid bacteria. However, the concentration and productivity of PLA have been low in previous studies. The enzymes responsible for conversion of phenylpyruvic acid (PPA) into PLA are equivocal.

**Methodology/Principal Findings:**

A novel thermophilic strain, *Bacillus coagulans* SDM, was isolated for production of PLA. When the solubility and dissolution rate of PPA were enhanced at a high temperature, whole cells of *B. coagulans* SDM could effectively convert PPA into PLA at a high concentration (37.3 g l^−1^) and high productivity (2.3 g l^−1^ h^−1^) under optimal conditions. Enzyme activity staining and kinetic studies identified NAD-dependent lactate dehydrogenases as the key enzymes that reduced PPA to PLA.

**Conclusions/Significance:**

Taking advantage of the thermophilic character of *B. coagulans* SDM, a high yield and productivity of PLA were obtained. The enzymes involved in PLA production were identified and characterized, which makes possible the rational design and construction of microorganisms suitable for PLA production with metabolic engineering.

## Introduction

Phenyllactic acid (PLA) has broad and effective antimicrobial activity against both bacteria and fungi and can therefore be employed and developed as a new type of natural antiseptic agent to extend the shelf life of food and feed [1]–[3]. It is also a useful precursor for the synthesis of many important drugs, including Danshensu (3,4-dihydroxyphenyllactic acid) which can inhibit platelet aggregation and coronary artery disease, hypoglycemic reagents, protease inhibitors, and anti-HIV reagents [4]–[8]. Because of its wide use in food and pharmaceutical industries, PLA production has attracted the attention of biotechnologists.

Chemical and biotechnological routes have been developed for PLA production. The chemical transformation strategy has some disadvantages, including a complex technology route, excessive by-products, and environmental pollution [9], [10]. Regarding the requirement for environmental protection and sustainable development, biotransformation has emerged as a powerful strategy for the production of this valuable compound [11]. Recently, several microorganisms, including *Geotrichum candidum*, propionibacteria, and lactic acid bacteria (LAB) were found to be PLA producers [12]–[18].

Many studies have focused on the ability of LAB to produce PLA because LAB have GRAS (generally recognized as safe) status. LAB, especially *Lactobacillus* strains, yielded PLA at the low level of 0.05–0.57 mM via the phenylalanine (Phe) metabolic pathway [19], [20]. The transamination reaction in the Phe pathway was the bottleneck for PLA formation [20], and the use of phenylpyruvic acid (PPA) as a substitute substrate led to a 14-fold increase in PLA production [21]. PPA showed obvious inhibitory effects in the biotransformation process, and therefore, fed-batch fermentation could be conducted for producing a high amount of PLA. It should be noted that PPA powder must be dissolved beforehand because of its slow dissolution rate at the biotransformation temperature [21]. PLA concentration was significantly increased with the application of this strategy [22].

PLA is the acknowledged reduction product of PPA, but the enzymes responsible for this reaction remain unclear. In previous studies, hydroxyisocaproate dehydrogenase (HicDH), phenyllactic acid dehydrogenase (PLDHase), and d-lactate dehydrogenase (d-LDH) of *Lactobacillus* were assumed to be involved in PLA production from PPA [19], [23]–[26].

In this study, a thermophilic strain, *Bacillus coagulans* SDM, was isolated and its ability to produce PLA from PPA was confirmed. The enzymes involved in the production of PLA were identified and characterized. In addition, with the enhanced solubility and dissolution rate of PPA at the high reaction temperature, a high concentration, yield and productivity of PLA were obtained in fed-batch bioconversion.

## Results

### Isolation of thermophilic bacteria for PLA production

Using the procedures described in Materials and Methods, 560 bacterial strains that grew at 50°C were tested for PLA production. Strain SDM showed the highest PLA production rate among the isolates and was selected for a detailed study. Identification of the strain was confirmed by Deutsche Sammlung von Mikroorganismen und Zellkulturen GmbH (DSMZ). The pattern of the fatty acid analysis was typical for *Bacillus* and suggested *B. coagulans*. Identification of the strain SDM as *B. coagulans* was also supported by the partial 16S rRNA sequence (GenBank accession number: HQ171055). The strain was deposited at the China Center for Type Culture Collection (CCTCC NO: M 2010012).

### Optimization of biotransformation by whole cells of *B. coagulans* SDM

**Figure 1:**
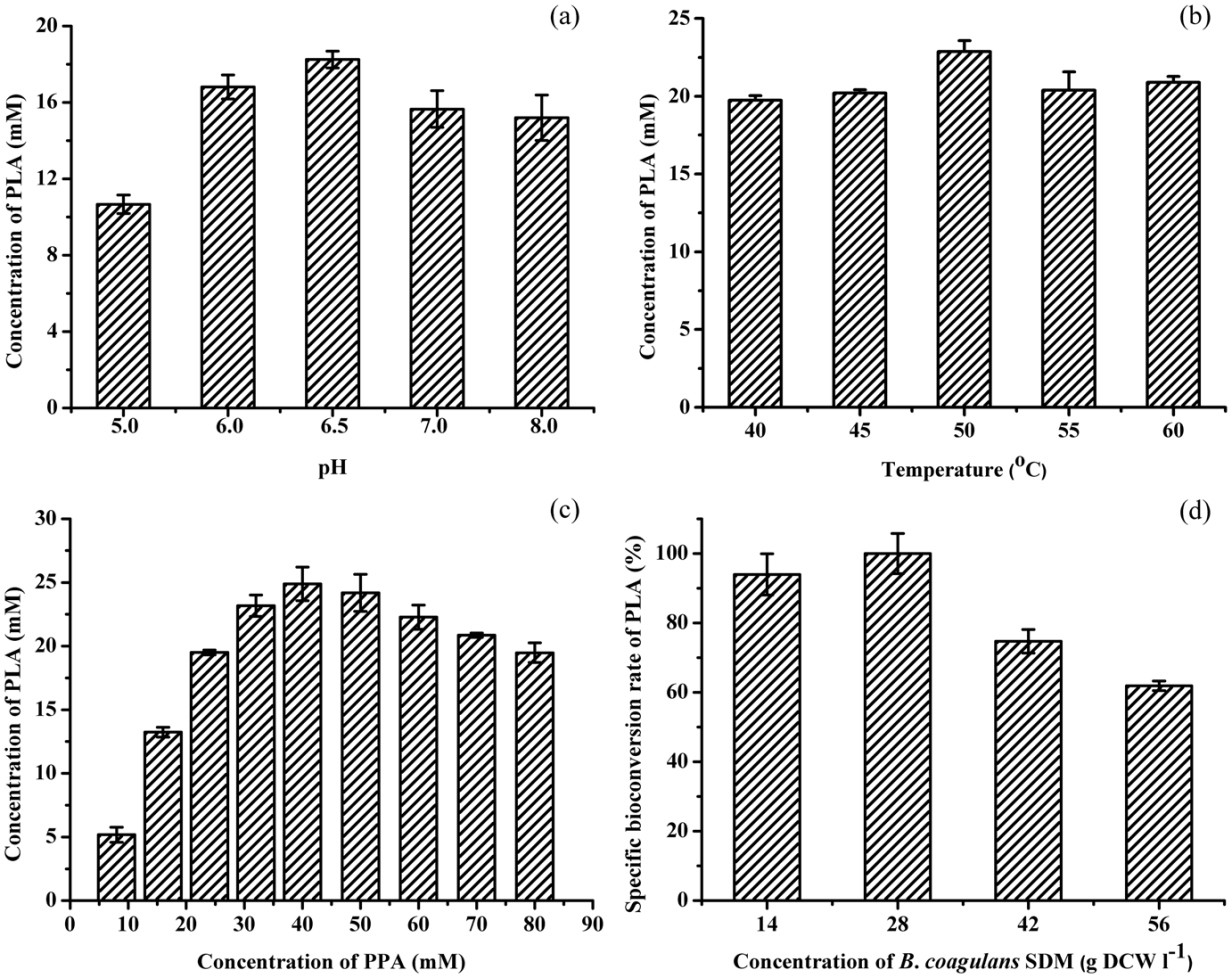
Optimization of bioconversion conditions. (a) Effect of pH on PLA production. (b) Effect of temperature on PLA production. (c) Effect of PPA concentration on PLA production. (d) Effect of biomass concentration on the specific bioconversion rate of PLA.

By using the thermophilic property of *B. coagulans* SDM, bioconversion of PLA from PPA was studied at a relatively high temperature. The reaction was carried out at 100 rpm for 0.5 h in phosphate buffer solution (PBS, 1/15 M [pH 7.4]) containing whole cells of *B. coagulans* SDM, PPA, and glucose. The optimal pH value was determined to be 6.5 (Figure 1a) and the optimal temperature was determined to be 50°C (Figure 1b). The optimal PPA concentration was determined to be 40 mM (Figure 1c) and substrate inhibition was observed at higher PPA concentrations. The effect of biomass concentration on PLA production was investigated and the highest specific bioconversion rate of PLA was obtained with 28 grams dry cell weight (DCW) per liter (OD_620 nm_ = 50) (Figure 1d). The effect of the cell growth phase on PLA production was also optimized and the middle exponential phase showed the highest PLA production rate (Table 1).

**Table 1:** Effects of cell growth phase on PLA production^a^.

Growth phase	Volume of collected broth (ml)	Reaction time (h)	Production of PLA (mg)	Bioconversion ability (mg h^−1^ per ml broth)
Early exponential phase	25	0.5	26.4±1.2	1.27±0.06
Middle exponential phase	25	0.5	47.2±1.8	2.27±0.09
Late exponential phase	25	0.5	19.4±0.2	0.93±0.01
Stationary phase	25	0.5	17.4±0.8	0.84±0.04

a Cells were cultivated to different growth phases and 25 ml broth of different phases were harvested, washed, and resuspended to form 10 ml cell suspensions, respectively. Reactions were carried out in 50-ml flasks containing 10 ml of the reaction mixtures for 0.5 h, and then the concentrations of PLA were determined.

### Production of PLA from PPA with substrate feeding

Fed-batch bioconversion was conducted with intermittent substrate feeding to avoid substrate inhibition. The bioconversion process was performed in a 5-l bioreactor (BIOSTAT B, B. Braun Biotech International GmbH, Germany) containing 2-l reaction mixtures. PPA was added in the solid form because of the observed enhanced dissolution of PPA at the high temperature. The initial concentrations of PPA and glucose were 6.6 g 1^−1^ (40 mM) and 36 g 1^−1^, respectively. The pH was maintained at 6.5 by addition of NaOH solution. PPA and glucose powders were supplemented to maintain the initial concentrations by a pulse-feeding strategy.

**Figure 2:**
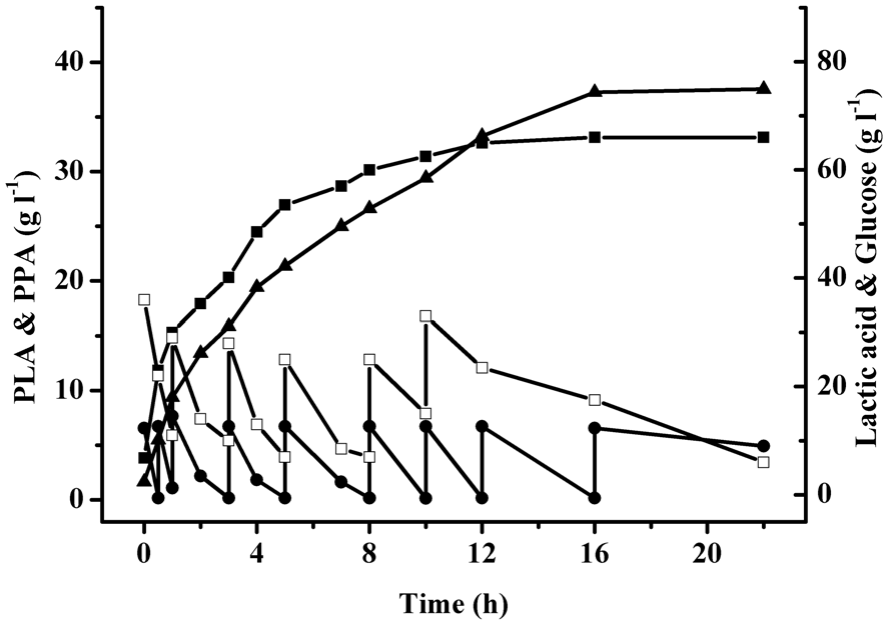
Time course of production of PLA from PPA under optimum conditions. Symbols represent: ▴, PLA; •, PPA; ▪, lactic acid; □, glucose.

The time course of PLA production from PPA was shown in Figure 2. A total of 105 g PPA and 252 g glucose were added to the 2-l reaction mixtures in this experiment. In addition to PLA, lactic acid was co-produced throughout the bioconversion process. The final PLA and lactic acid concentrations were 37.3 g 1^−1^ and 66 g 1^−1^, respectively. The average productivity of PLA was 2.3 g 1^−1^ h^−1^ and the yield was 70%.

### Identification of enzymes responsible for conversion of PPA into PLA

**Figure 3:**
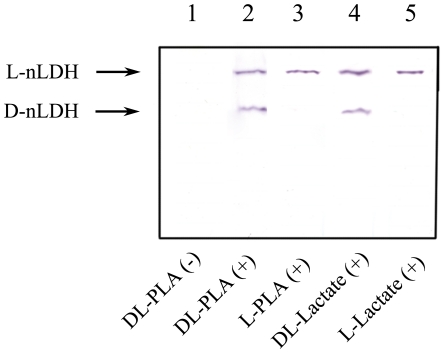
Native polyacrylamide gel electrophoresis staining for PLA and lactate oxidation activities. Symbol (−) represents solutions without NAD addition and symbol (+) represents solutions with 5 mM NAD. dl-PLA was used as substrate for active staining in lane 1 and 2, whereas l-PLA, dl-lactate, and l-lactate were used as substrates in lane 3, 4, and 5, respectively. All substrates were used at a concentration of 50 mM.

The conversion between PPA and PLA is a redox reaction. To identify the enzymes responsible for the production of PLA, activity staining of the enzymes that catalyzed PLA oxidation was carried out. As shown in Figure 3 (lane 1), no PLA oxidation activity was detected without NAD addition. In the presence of NAD, two bands with distinct mobilities were presented when dl-PLA was used as the substrate (Figure 3, lane 2). The activities responsible for l- and d-lactate oxidation were also studied because of the co-production of lactic acid throughout the bioconversion process. The two bands exhibiting l- and d-lactate oxidation activities were located at the same positions as the two enzymes involved in l- and d-PLA oxidation, respectively (Figure 3). Since the biotransformation of lactic acid from pyruvic acid is catalyzed by the NAD-dependent lactate dehydrogenases (nLDHs), the results of activity staining implied that the enzymes responsible for PPA reduction present in *B. coagulans* SDM are nLDHs.

### Activities of l-nLDH and d-nLDH in PPA reduction

To further identify the function of nLDHs in PLA production activities in strain SDM, the nLDHs were purified and further characterized. At present, only one genome of the *B. coagulans* strain, *B. coagulans* 36D1, has been released. Analysis of the genome of *B. coagulans* 36D1 identified one l-nLDH (accession number at GenBank, ZP_04430752) and one d-nLDH (accession number at GenBank, ZP_04430367). By using these two genes as guides for designing primers, the *ldh*L gene (GenBank accession number: HQ148709) and *ldh*D gene (GenBank accession number: HQ148710) of *B. coagulans* SDM were cloned.

The amino acid sequences of the cloned nLDHs were deduced from the nucleotide sequences, and l-nLDH and d-nLDH exhibited considerable homology with other reported l-nLDHs and d-nLDHs of LAB, respectively (Figure S1 and Figure S2). Multiple alignments with other reported l-nLDHs showed that all catalytically important residues (Arg 109, Asp 168, Arg 171, and His 195) were conserved in the *B. coagulans* SDM l-nLDH (Figure S1) [27]. Moreover, the amino acids involved in activation by fructose 1,6-bisphosphate (FDP) (Arg 173 and His 188) were also found in l-nLDH (Figure S1) [28]. The essential residues of d-nLDH (Arg 235, Glu 264, and His 296) were also conserved in the *B. coagulans* SDM d-nLDH (Figure S2) [29], [30].

The catalytic efficiencies of the recombinant nLDHs were determined by the reduction of pyruvate or PPA. Although notable differences in the catalytic activities were observed, both pyruvate and PPA could be catalyzed by the l-nLDH and d-nLDH of *B. coagulans* SDM (Table 2). The catalytic efficiencies of l-nLDH and d-nLDH on pyruvate were 40-fold and 3-fold higher than on PPA, respectively. A possible explanation is that PPA has a larger group at the C3-position, which results in a substrate that is unfavorable compared with pyruvate [31]–[33].

**Table 2:** Kinetic parameters of l-nLDH and d-nLDH for pyruvate and PPA.

Substrate	l-nLDH				d-nLDH			
	*K*_m_ (mM)	*V*_max_ (U/mg)	*k*_cat_ (s^−1^)	*k*_cat_/*K*_m_ (M^−1^ s^−1^)	*K*_m_ (mM)	*V*_max_ (U/mg)	*k*_cat_ (s^−1^)	*k*_cat_/*K*_m_ (M^−1^ s^−1^)
Pyruvate	2.6	1800.5	1172.2	4.5×10^5^	2.2	33.7	23.6	1.1×10^4^
PPA	4.3	72.6	47.2	1.1×10^4^	4.4	23.5	16.5	3.9×10^3^

## Discussion

In previous studies, PPA as the direct precursor of PLA was used as a substitute for Phe to enhance PLA production [21]. PPA is difficult to dissolve at a low temperature or under acidic and neutral conditions. This problem was solved by dissolving PPA with 2 M NaOH beforehand and adjusting the pH of the media by acid after the PPA solution was added [21]. In this study, a *B. coagulans* strain that transformed PPA to PLA efficiently at 50°C was isolated. PPA powder was directly added to the reaction mixture by using a pulse-feeding strategy in the fed-batch bioconversion. The rapid dissolution of PPA and the high metabolic activity at higher temperature resulted in a higher concentration and yield of PLA in *B. coagulans* SDM compared with other reported producers (Table 3).

**Table 3:** Comparison of PLA production by bacteria.

Organism	Substrate	Temperature (°C)	Time (h)	Production process	Concentration (g l^-1^)	Yield	Reference
*Lactobacillus plantarum* ITM21B	Phe	30	72	-	0.055	0.14	[18]
*Lactobacillus plantarum* TMW1.468	Phe	30	48	-	0.12	0.14	[20]
*Lactobacillus sanfranciscensis* DSM20451^T^	Phe	30	48	-	0.042	0.05	[20]
*Lactobacillus* sp. SK007	Phe	30	72	Batch	0.061	0.06	[21]
*Lactobacillus* sp. SK007	PPA	30	24	Batch	0.86	0.85	[21]
*Lactobacillus* sp. SK007	PPA	30	72	Fed-batch	17.38	0.511	[22]
*B. coagulans* SDM	PPA	50	16	Fed-batch	37.3	0.70	This study

The enzymes responsible for PPA reduction were uncertain in previous studies. HicDH was shown to catalyze the reduction of aromatic α-keto acids to the corresponding hydroxyl acids in the amino acid catabolism of many LAB [26]. In some studies, it was assumed that PLDHase was responsible for reducing PPA to PLA during Phe catabolism in LAB [23], [25]. Recently, a d-LDH from the PLA producing strain, *Lactobacillus* sp. SK007, was purified and its reducing activity on PPA was detected [19]. In this study, the enzymes responsible for PPA reduction were identified as l-nLDH and d-nLDH through enzyme activity staining and kinetic analysis.

**Figure 4:**
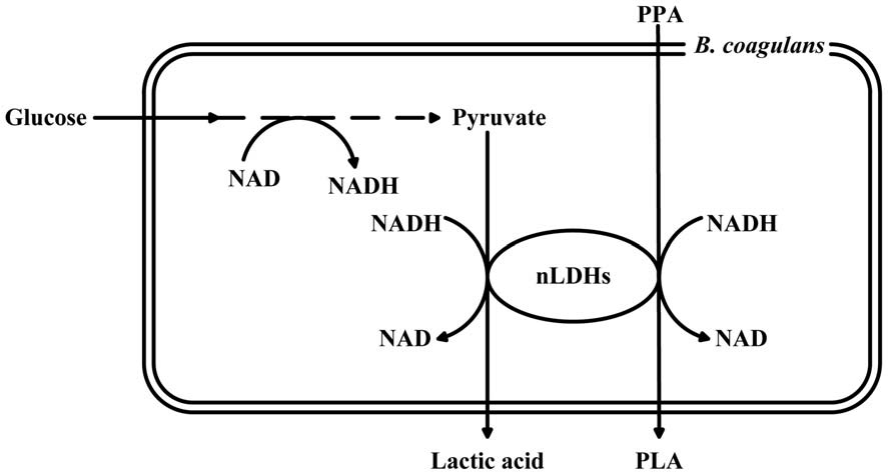
Schematic view of PLA production system of *B. coagulans* SDM.

A cosubstrate was necessary to supply NADH because conversion of PPA to PLA was accompanied by the oxidation of NADH to NAD. In general, glucose is a good carbon source for cofactor regeneration because of its commercial availability, low price, and excellent bioavailability. Therefore, glucose was used for cofactor regeneration in this study. The biotransformation was conducted as follows: NADH was produced from glycolysis and PPA was reduced to PLA via nLDH-catalyzed reduction (Figure 4). Because both pyruvate and PPA are the substrates of nLDHs (Figure 4), the pyruvate derived from glucose would be expected to compete against PPA for binding to nLDHs and produce a mass of lactic acid (Figure 2). For a substrate such as PPA with larger groups at the C3-position, the catalytic efficiencies of nLDHs were markedly decreased (Table 2) [31]–[33]. Overcoming this bottleneck by redesign of the wild-type enzymes would significantly improve PLA production.

In conclusion, PLA is efficiently produced from PPA by the novel thermophilic strain *B. coagulans* SDM. In fed-batch bioconversion, 37.3 g 1^−1^ PLA was obtained from 52.5 g 1^−1^ PPA in 16 h at 50°C. The enzymes responsible for PPA reduction were further examined and identified as l-nLDH and d-nLDH. Illustrating this problem makes it possible to rationally modify microorganisms that are suitable for PLA production through metabolic engineering.

## Materials and Methods

### Isolation of thermotolerant bacteria

Soil samples were collected from chemical plants, farmlands, gardens, and dust heaps. Approximately 1 g of each sample was enriched in 50 ml of a liquid medium (GYSC) containing (g l^-1^): glucose (50), yeast extract (10), soybean peptone (5), and CaCO_3_ (20). The samples were incubated at 50°C and 100 rpm for 8 h, and an aliquot of the suspension was plated on GYSC solid medium and incubated at 50°C until multiple colonies emerged. Representative colonies were selected and incubated in GYSC liquid medium at 50°C and 100 rpm. After 8 h, 1% PPA (w/v) was added to the medium. The reaction mixtures were incubated for another 8 h, centrifuged at 4,500 rpm for 15 min, and the supernatant was analyzed for PPA and PLA. The strain that produced the highest amounts of PLA was designated SDM.

### Biotransformation by whole cells of strain SDM

For whole cell preparation, overnight precultures of strain SDM were inoculated into fresh GYSC liquid medium with 10% inoculum and the cultures were incubated at 50°C and 100 rpm. Cells were harvested by centrifugation at 4,500 rpm for 15 min, washed with PBS, and resuspended in the same buffer to form cell suspensions. The biotransformation reactions were performed in 50-ml flasks containing 10 ml of the reaction mixtures. Parameters varied as follows for optimization of the reaction conditions: pH values were 5–8. Temperatures were 40–60°C. PPA concentrations were 8–80 mM, and cell concentrations were 14–56 g DCW 1^−1^. The time of harvesting cells was set at early exponential phase, middle exponential phase, late exponential phase, and stationary phase. After completion of the reactions, the samples were heated to 100°C and centrifuged. The concentrations of PPA and PLA in the resulting supernatants were quantitatively analyzed by high-performance liquid chromatography (HPLC).

### Activity staining after native polyacrylamide gel electrophoresis (PAGE)

To identify the enzyme activities for PLA production in *B. coagulans* SDM, cells were harvested, washed, resuspended in PBS, and disrupted by sonication in an ice bath. The disrupted cells were centrifuged for 40 min at 13,000 rpm and the supernatant was used as the crude cell extract for native PAGE. Native PAGE was carried out at 4°C on a 10% native polyacrylamide gel. Active staining was performed after native PAGE according to a previous method with some modifications [34]. The gel was cut into five parts after electrophoresis and soaked in different solutions containing 100 mM Tris-HCl buffer (pH 8.3), 0.15 mM phenazine methanosulfate (PMS), 0.15 mM nitro blue tetrazolium (NBT), and corresponding substrates. Chemically reduced NBT dye by enzymatically produced NADH was precipitated as a dark blue band that was easily seen in the stained gels.

### Bacterial strains, plasmids, and growth conditions

*Escherichia coli* strains and plasmids used in this study are listed in Table 4. *E. coli* was grown at 37°C in Luria-Bertani (LB) medium and kanamycin was added at a concentration of 50 µg m1^−1^, if necessary.

**Table 4:** Strains, plasmids, and primers used in this work.

Strain, plasmid, or primer	Relevant characteristics	Source or reference
Strains		
*E. coli* DH5α	φ80 *lacZ*ΔM15 Δ(*lacZ*YA-*arg*F) U169 *recA*1 *endA*1 *hsdR*17 *supE*44λ- *thi*-1	Novagen
*E. coli* BL21(DE3)	*F^−^ ompT gal dcm lon hsdS*_B_(r_B_^-^m_B_^-^) λ(*DE3*)	Novagen
Plasmids		
pET-28a	Expression vector, Kan^r^	Novagen
pET28a-*ldh*L	N and C-terminal His-tagged *ldh*L in pET-28a	This study
pET28a-*ldh*D	N and C-terminal His-tagged *ldh*D in pET-28a	This study
Primers		
P1	5′-CCGC*AAGCTT*CGATGAAAAAGGTCAATCGTATTGCAG-3′	This study
P2	5′-TTAA*CTCGAG*CAATACAGGTGCCATCGTTTCT-3′	This study
P3	5′-GCGC*AAGCTT*CGATGAGAAAAGTTGTTGCCTA-3′	This study
P4	5′-CTAA*CTCGAG*TGATTTTATCTCCCACCTGC-3′	This study

The italic face indicates the introduction of restriction sites.

### Recombinant protein production and purification

The *ldh*L and *ldh*D genes were amplified by PCR using genomic DNA of *B. coagulans* SDM as the template. Primers were designed based on the genome sequences of *B. coagulans* 36D1. Primers P1 and P2 were used for *ldh*L amplification and primers P3 and P4 were used for *ldh*D amplification (Table 4). The resulting PCR products were digested with *Hin*dIII-*Xho*I and cloned into the *Hin*dIII-*Xho*I sites of pET-28a to construct plasmids pET28a-*ldh*L and pET28a-*ldh*D. For protein expression, *E. coli* BL21(DE3) harboring recombinant plasmid was incubated in LB media (containing 50 µg m1^−1^ kanamycin) at 37°C with shaking. When the culture reached an optical density of 0.6 at 600 nm, 1 mM isopropyl-β-d-thiogalactopyranoside (IPTG) was added to induce gene expression. After induction at 25°C for 5 h, cells were harvested and suspended in the binding buffer (200 mM sodium phosphate, 500 mM sodium chloride, and 20 mM imidazole [pH 7.4]) and disrupted by sonication. The suspension was centrifuged to remove cell debris. The resultant supernatant was filtered and loaded onto a HisTrap HP 5 ml column (GE Healthcare) and the enzyme was eluted with 60% binding buffer and 40% elution buffer (200 mM sodium phosphate, 500 mM sodium chloride, and 500 mM imidazole [pH 7.4]) at a flow rate of 5 ml min^−1^. The fractions containing the enzyme were detected by sodium dodecyl sulfate-polyacrylamide gel electrophoresis (SDS-PAGE). The protein concentration was determined by the Bradford method using bovine serum albumin for calibration [35].

### Lactate dehydrogenase assay

Reduction activities of nLDHs on pyruvate or PPA were assayed at 50°C with freshly purified recombinant l/d-nLDHs. For the d-nLDH assay, the reaction mixture contained 50 mM Tris-HCl buffer (pH 7.0), 0.2 mM NADH, and 10 mM substrate. For the l-nLDH assay, the catalytic reaction mixtures contained all reagents mentioned above in addition to 5 mM FDP. One unit of l/d-nLDHs was defined as the amount that catalyzed the oxidation of 1 µmol of NADH per minute.

### Analytical procedures

PPA and PLA were measured by HPLC (Agilent 1100 series, Hewlett-Packard, USA) equipped with an Agilent Zorbax SB-C18 column (150×4.6 mm, 5 µm) and a variable-wavelength detector at 210 nm. The mobile phase consisted of 1 mM H_2_SO_4_ and acetonitrile with a ratio of 85:15 (v/v) at a flow rate of 0.7 ml min^−1^ at 30°C.

Glucose and lactic acid concentrations were measured by HPLC equipped with a Bio-Rad Aminex HPX-87H column (300×7.8 mm) and a refractive index detector. Analysis was performed with a mobile phase of 10 mM H_2_SO_4_ at a flow rate of 0.4 ml min^−1^ at 55°C.

DCW was calculated from the optical density (OD_620 nm_) with a linear correlation factor (1 OD_620 nm_ = 0.56 g DCW 1^−1^).

## Supporting Information

Figure S1 **Multiple alignment of the amino acid sequences of l-nLDHs from selected species.** Species and accession numbers of sequences are as follows: *Lactobacillus casei* ATCC 393 (Lcas), YP_001988625; *Lactobacillus rhamnosus* GG (Lrha), YP_003172269; *Lactococcus lactis* (Llac), AAB51674; *Lactobacillus helveticus* DPC 4571 (Lhel), YP_001576807; and *B. coagulans* SDM (Bcoa), this study.
(TIF)

Figure S2 **Multiple alignment of the amino acid sequences of d-nLDHs from selected species.** Species and accession numbers of sequences are as follows: *Lactobacillus johnsonii* NCC 533 (Ljoh), NP_964061; *Lactobacillus helveticus* DSM 20075 (Lhel), ZP_05752035; *Leuconostoc mesenteroides* (Lmes), AAA99506; *Lactobacillus pentosus* ATCC 8041 (Lpen), BAA14352; and *B. coagulans* SDM (Bcoa), this study.
(TIF)
