# Supplementary figures and images for: Efficient Conversion of Phenylpyruvic Acid to Phenyllactic Acid by Using Whole Cells of Bacillus coagulans SDM
Source: PLoS One. 2011 Apr 20;6(4):e19030. doi: 10.1371/journal.pone.0019030 (PMC3080406; doi:10.1371/journal.pone.0019030)

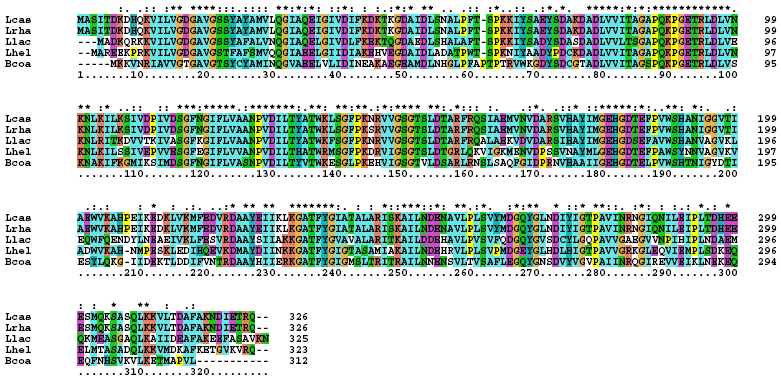

Supplement: Figure S1 — Multiple alignment of the amino acid sequences of l-nLDHs from selected species. Species and accession numbers of sequences are as follows: Lactobacillus casei ATCC 393 (Lcas), YP_001988625; Lactobacillus rhamnosus GG (Lrha), YP_003172269; Lactococcus lactis (Llac), AAB51674; Lactobacillus helveticus DPC 4571 (Lhel), YP_001576807; and B. coagulans SDM (Bcoa), this study. (TIF) [file pone.0019030.s001.tif]

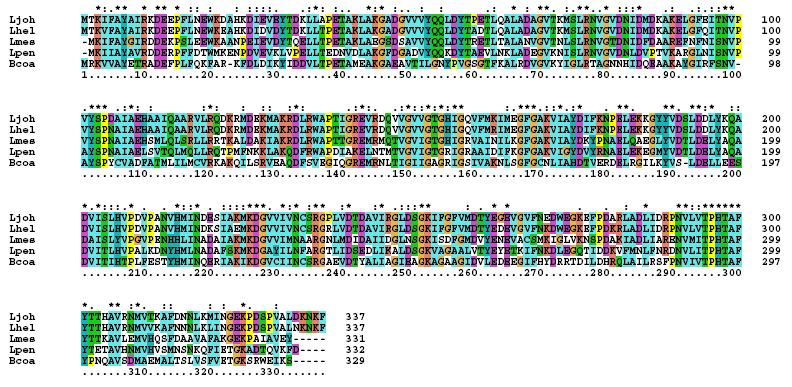

Supplement: Figure S2 — Multiple alignment of the amino acid sequences of d-nLDHs from selected species. Species and accession numbers of sequences are as follows: Lactobacillus johnsonii NCC 533 (Ljoh), NP_964061; Lactobacillus helveticus DSM 20075 (Lhel), ZP_05752035; Leuconostoc mesenteroides (Lmes), AAA99506; Lactobacillus pentosus ATCC 8041 (Lpen), BAA14352; and B. coagulans SDM (Bcoa), this study. (TIF) [file pone.0019030.s002.tif]
